# Thoracic duct lymphangioma and chylous ascites in a 24-year-old female

**DOI:** 10.4322/acr.2021.290

**Published:** 2021-05-27

**Authors:** Larissa Moreira Galvão Bello, Vitorino Modesto dos Santos, Márcia Mayumi Marques Suzuki, Naiara Regina Siqueira, Fernanda Gonçalves Reis

**Affiliations:** 1 Hospital das Forças Armadas (HFA), Departamento de Medicina, Brasília, DF, Brasil; 2 Universidade Católica de Brasília, Curso de Medicina, Brasília, DF, Brasil

**Keywords:** Chylous Ascites, Lymphangioma, Octreotide, Thoracic Duct, Treatment

## Abstract

Lymphangiomas are rare and correspond to 0.7% to 4.0% of mediastinal tumors, and isolated mediastinal location occurs in 1% of cases. They are benign tumors that originate from a congenital malformation of the lymphatic vessels and are diagnosed more frequently in children less than 2 years of age. Chylous ascites is a clinical manifestation of thoracic duct lymphangioma and is composed of lymph accumulation caused by dilation of this lymphatic channel. It appears milky in the peritoneal cavity, containing triglyceride levels higher than 200 mg/dl. We report the case of a young patient with chylous ascites and lymphangioma of the thoracic duct, who was conservatively treated with octreotide and a low-fat diet with medium-chain triglycerides.

## INTRODUCTION

Lymphangiomas are tumors originating from the abnormal development of lymphatic vessels, which impede the normal flow of lymph, forming cysts lined with vascular endothelium. They are congenital malformations, and more than 80% of patients are below 5 years age.^1^ ^,^ ^2^ They can occur in any region of the body, most commonly in the head and neck, and show progressive development, which can generate compressive symptoms and invasion of adjacent structures, with a clinical picture compatible with their location^3^ Chylous ascites is a clinical manifestation of thoracic duct lymphangioma due to lymph exudate caused by the dilation of this lymphatic channel. The ascitic fluid presents the gross aspect of a milky-like secretion accumulated in the peritoneal cavity, with triglycerides cutoff value of > 200 mg/dL.^4^ ^-^ ^7^ As in the present case, the mechanism of ascites might be the result of a direct loss of chylous fluid through a lymphatic-peritoneal fistula, associated with the portal or non-portal etiologies, including either congenital malformations or acquired conditions.^4^ ^-^ ^7^

We report the case of a woman with thoracic duct lymphangioma, which was investigated through chylous ascites that the patient initially presented, with a brief review of the clinical aspects, diagnosis, and treatment of this pathology. The aim of the study is to report the case of a young patient with chylous ascites due to thoracic duct lymphangioma who was treated conservatively with octreotide and a low-fat diet with medium-chain triglycerides (TCM). In addition to increasing the index of suspicion of this pathology so that the patients can be treated as earliest as possible.

## CASE REPORT

**Figure 1:**
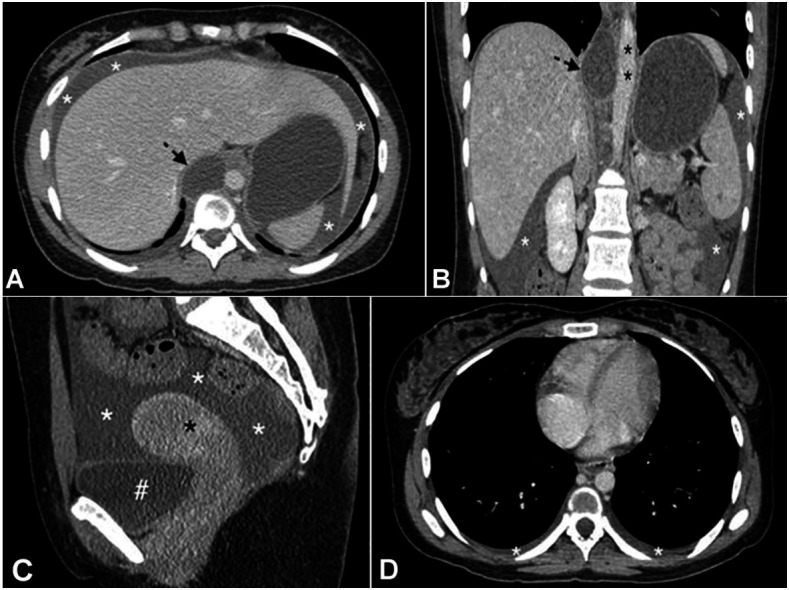
Thoracic and abdominal enhanced computed tomography. **A –** axial plane and **B** coronal plane – images at admission showing a cystic image (black arrow) in the thoracic duct site at the thoracic-abdominal transition, well-defined and without enhancement in contact with the aorta (black asterisk), and associated with ascites (white asterisks), **C –** sagittal plane and **D** axial plane showing ascites (white asterisks) and bilateral laminar pleural effusion, as well as the bladder (**#**) and the uterus (black asterisk).

A previously healthy 24-year-old Afro-descent woman was admitted with pain in the upper abdomen that radiated to the back, of high intensity (8/10) and constant, worsening on inspiration and at dorsal decubitus, improving with analgesics and associated with a high fever (39 ^o^C) which started four days after admission. She denied drug allergies, smoking, alcoholism, abdominal trauma, and a family history of cancer. She had been using oral contraceptives for three years and had excised a benign nodule in the right breast. Physical examination revealed a diffuse pain on deep palpation and abdominal distension. Laboratory tests revealed normal hematocrit (14.6%); 7750 leukocytes with 5% band forms; C-reactive protein: 23.8 mg/dL; and beta HCG less than 0.1 mIU/ml. Serologies for HIV, HTLV, viral hepatitis, syphilis, *Chlamydia trachomatis*, Chagas disease, and toxoplasmosis were negative, and cytomegalovirus IgG positive and IgM negative. Urine and blood cultures were negative; negative tests for rheumatic activity; triglycerides (≤ 150): 186 mg/dL, HDL (> 60): 36 mg/dL, LDL: (≤ 129) 129.8 mg/dL, and total cholesterol: (≤ 200) 203 mg/dL. CA 15.3, CA 19.9, CA 72.4, and CEA were normal, but CA 125 (≥ 35): 330.6 U/mL. Contrast-enhanced abdominal computed tomography (CT) showed a well-delimited cystic image without enhancement, located in the topography of the thoracic duct in the thoracoabdominal transition and in contact with the aorta, associated with massive ascites and mild bilateral pleural effusion (Figures 1).

**Figure 2:**
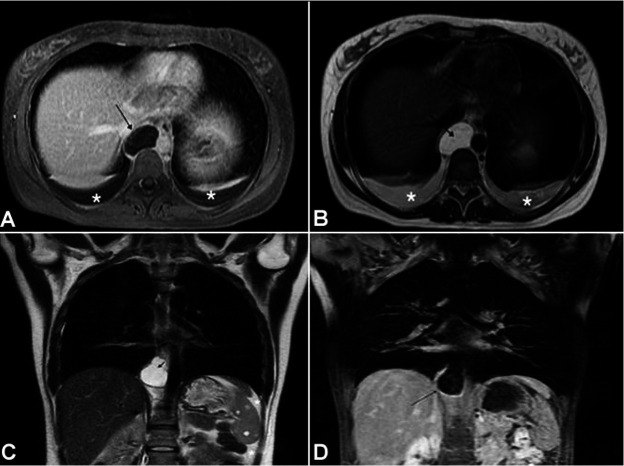
Axial MRI sections of the chest showing a cystic image in the topography of the thoracic duct (arrow) characterized by **A –** a low signal in the T1-weighted sequence; **B –** a high signal in the T2-weighted sequence; associated with **C –** small pleural effusion and ascites (asterisks); the cyst is also evident (arrow) in the coronal section on the T2-weighted sequence, and **D –** without enhancement in the T1-weighted sequence after contrast injection.

**Figure 3:**
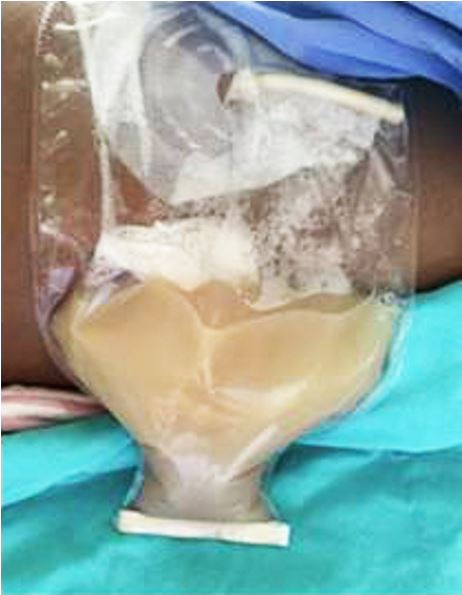
Gross view of the milky-like liquid collected on the drainage of the ascites.

Magnetic resonance imaging (MRI) of the chest with contrast revealed a cystic image at the thoracoabdominal transition, in the topography of the thoracic duct on the right of the aorta, measuring 4.0 cm x 3.8 cm x 2.2 cm, with homogeneous content and lobulated contours in the upper portion (Figure 2). The investigation for neoplasms with PET-CT and upper digestive endoscopy did not reveal the presence of malignancy. The patient underwent exploratory laparotomy through a Pfannenstiel incision, and approximately one liter of liquid with a milky aspect was drained (Figure 3), and a tubule-laminar drain was placed in the cavity.

**Figure 4:**
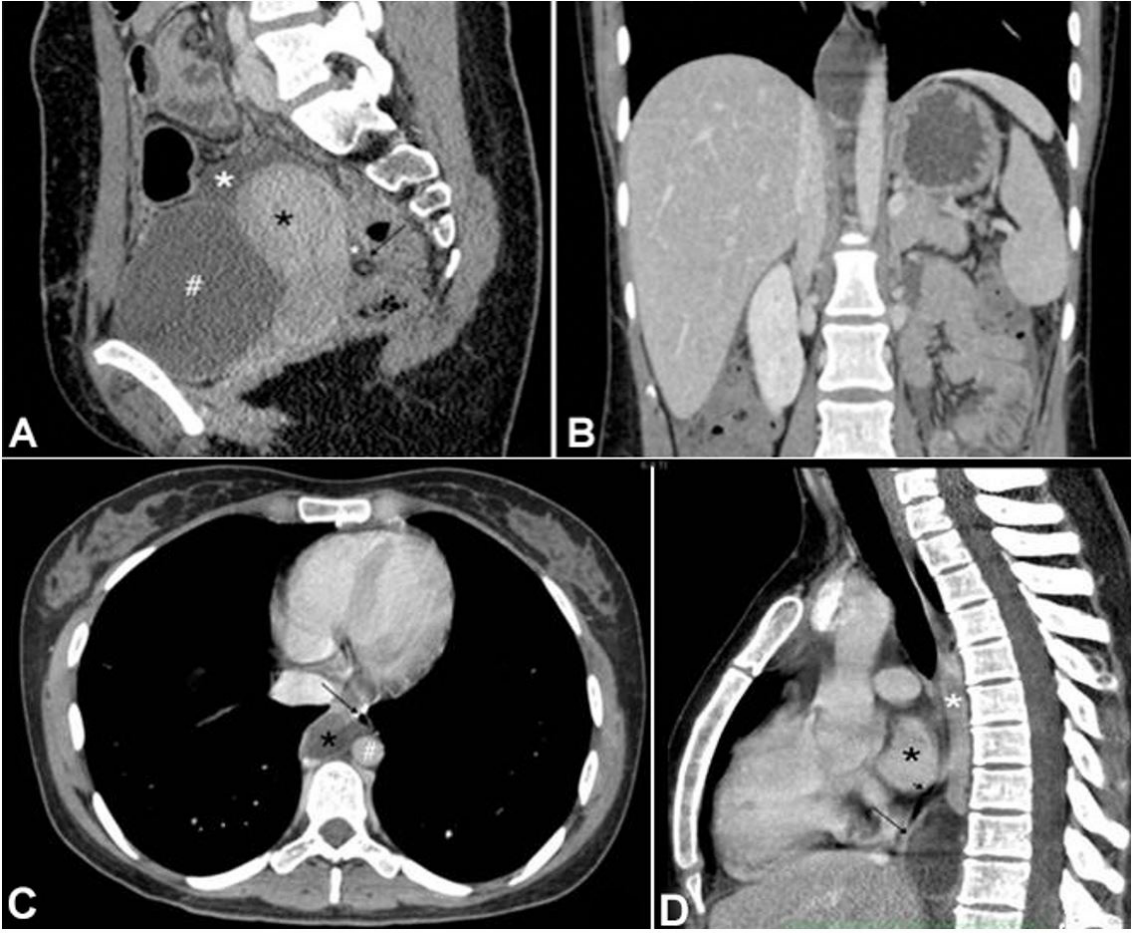
Contrast-enhanced CT scan of the abdomen after drainage, **A –** shows a significant reduction in ascites (white asterisk) and partially drainage (arrow) in the sagittal plane, in addition to the uterus (black asterisk) and the bladder (**#**); **B –** the coronal section image, confirms the total regression of ascites detected in the admission exam; **C** and **D –** Contrast-enhanced chest CT scan after drainage, C - axial section, and mediastinal window shows the close relationship between the cyst and the thoracic duct (black asterisk) and other structures such as the descending aorta (**#**), the azygous vein (white asterisk) and the esophagus (arrowhead); it also reveals the absence of pleural effusion; D - sagittal section shows the major diameter of the cyst at the site of the thoracic duct (long arrow), its location in the posterior mediastinum and the relationship with the esophagus (short arrow), the right ventricle (black asterisk), and the azygous vein (white asterisk).

The analysis of the liquid characterized chylous ascites containing mononuclear (65%), polymorphonuclear (31%), and eosinophils (4%); total proteins: 1.46 g/dL; glucose: 116.8 mg/dL; DHL: 93.7 U/L; triglycerides: 511.5 mg/dL; the culture showed *Staphylococcus lugdunensis*; and the search for fungi, mycobacteria, and oncotic cytology were negative. With a diagnosis of thoracic lymphangioma, the patient had a zero diet for 48 hours, an oral diet with medium-chain triglycerides for seven days, and was medicated with octreotide. There was a decrease in daily ascitic output in abdominal and chest fluid by CT control images (Figure 4), and clinical improvement.

There were no changes in the pulmonary parenchyma that had normal attenuation coefficients, and there was no lymph node enlargement in the mediastinum or in the lower cervical chain. With no flow, the abdominal cavity drain was removed, and the patient was discharged from the hospital in good general condition, with guidance for specialized monitoring in the outpatient service of Thoracic Surgery.

## DISCUSSION

Lymphangiomas are benign tumors originating from congenital malformations of lymphatic vessels and presenting as dilatations of variable volumes forming cysts that are lined with vascular endothelium, which impair the normal flow of lymph.^1^ ^-^ ^3^ They are rare, representing 0.7% to 4% of mediastinal tumors, and the isolated location at that site corresponds to 1% of cases. This pathology is three times more common in females, and 90% of diagnoses are established in childhood.^3^ The head and neck regions are the most affected in two-thirds of the cases.^8^ Abdominal lymphangiomas are very uncommon with a reported incidence of 1 in 20,000 to 250,000 population.^2^ The differential diagnosis of lymphangiomas includes cystic lesions of the mediastinum, such as thymic cyst, teratoma, hematomas, thyroglossal duct cyst, necrotic tumors and lymph nodes, meningomyelocele and pericardial or bronchogenic cysts.^3^ Lymphangiomas are usually asymptomatic, being incidentally discovered in adults. In children, there may be early compressive symptoms due to the reduced space for their growth. The occurrence of symptoms depends on the location, size, and speed of the lesion growing. Dyspnea is the most common, but hoarseness, respiratory failure, or superior vena cava syndrome can occur due to extrinsic compression, as well as chylothorax, chylopericardium, or chyloperitoneum. During infectious conditions, there may be acute symptoms caused by bleeding or excessive lymph production.^3^ The definitive diagnosis of lymphangioma depends on anatomopathological confirmation by evaluation of a surgical sample.^3^

Chylous ascites is also rare, with the incidence of hospital admissions estimated at approximately 1: 20,000 up to 1: 180,000, according to international studies.^5^ ^,^ ^6^ This is characterized by the accumulation of milky fluid in the abdominal cavity with a high level of triglycerides. The most common causes of ascites in the West are liver cirrhosis and malignancies in more than 60% of cases; less common are tuberculosis, filariasis, paracoccidioidomycosis, congenital, inflammatory, postoperative, and traumatic.^4^ ^-^ ^7^ ^,^ ^9^ This ascites can manifest with nausea, anorexia, early satiety, weight loss, abdominal distension, nonspecific abdominal pain, dyspnea, fever, and night sweats.^6^ The definitive diagnosis is made by paracentesis, with a milky-looking liquid and triglyceride dosage higher than 200 mg/dl. Associated with detailed anamnesis and physical examination, routine laboratory data including hypogammaglobulinemia, hypogammaglobulinemia, and deficiency of fat-soluble vitamins when there is a large amount of ascitic fluid, can corroborate for the diagnosis. Radiological studies such as CT and MRI are useful in identifying the presence of free fluid in the cavity, pathological masses, and lymph node enlargement; while lymphangiography and lymphoscintigraphy can help to detect dilation of lymphatic vessels, fistulas, and the permeability of the thoracic duct.^5^ Treatment of chyloperitoneum can be either conservative or surgical; the latter is indicated when there is no improvement with conservative treatment in two to three weeks.^8^ Cases that maintain drainage volumes above 1000 mL per day for more than five days are considered non-responsive.^4^ ^,^ ^6^ ^,^ ^7^ ^,^ ^9^ The conservative approach consists of a low-fat, high-protein, and TCM-rich diet, in addition to diuretics and octreotide. Restriction of long-chain triglycerides prevents the production of monoglycerides and free fatty acids that are transported as chylomicrons to the intestinal lymphatic ducts. The medium-chain TCM-rich diet reduces the production of lymph, as these triglycerides are absorbed directly into the portal vein system.^7^ The oral route should be a priority option, but the parenteral diet starts when there is no response to the oral.^5^ Associated with dietary treatment, octreotide, a somatostatin analog is used, which increases splanchnic arteriolar resistance by inhibiting the secretion of pituitary and gastrointestinal hormones, with the reduced lymphatic flow as a result of decreased gastrointestinal flow. Studies have shown that the use of octreotide can decrease the number of surgical interventions and reduce the treatment period.^6^ ^,^ ^9^ ^,^ ^10^ Worthy of note was the improvement from 600 mL to 25 mL per day of chylous fluid, collected by peritoneal catheter, in a woman with liver cirrhosis and uncontrolled recurrent ascites. With a poor prognosis, she utilized octreotide 100 mcg three times daily for five days.^6^ Octreotide reduces complications of chylous ascites due to lymphatic fluid loss causing hypoalbuminemia, coagulation, hydro electrolytic, and immunological disorders, which result in impairment of general clinical status and increased morbidity and mortality.^6^ ^,^ ^9^ ^,^ ^10^

The peritoneal fluid analysis showed classical chylous ascites without infective features; nevertheless, some bacterial role in the thoracic duct change was not discarded. *S. lugdunensis* infections are infrequent, and the knowledge about clinical characteristics is lacking. Research is necessary to better clear the diversity and virulence of this species and its role in urinary and respiratory tract infections, peritonitis, and bacteremia.^11^ The agent was identified in the ascitic fluid more than 48h after admission to the hospital. Notwithstanding, the present case was not considered as a healthcare-associated infection because the patient presented fever and pain in the thoracoabdominal transition before admission. An initial concern was on an undetected bone or joint infection, bacteremia, and peritonitis, but blood cultures were negative.^11^

The present report is in agreement with the literature because it is a young patient without suspicion of a congenital entity, and asymptomatic before the current hospitalization. The initial manifestation that led to the consultation was a consequence of chylous ascites, and resulted in the diagnosis of thoracic duct lymphangioma as the etiology of chyloperitoneum. The characteristics of this lymphangioma as shown in imaging exams, are of a single mediastinal cyst, a rare variety among mediastinal tumors. As the treatment instituted was conservative, there was no anatomopathological study. With the possibility of the progressive growth of lymphangiomas over the years, the patient will remain under regular outpatient monitoring for periodic imaging control.

## CONCLUSION

Chylous ascites associated with thoracic duct lymphangioma is a therapeutic challenge even currently, as this is a very rare condition that is related to elevated morbidity. The authors believe that case studies can increase the index of suspicion in the first manifestation of ascites allowing that diagnosis and control be established early.

## References

[1] Salehi F, Landis M, Inculet R, Wiseman D (2019). Case report of a rare cystic mediastinal lymphangioma mimicking recurrent pleural effusion. Case Rep Radiol.

[2] Hamaguchi Y, Arita S, Sugimoto N (2020). Laparoscopic resection of abdominal cystic lymphangioma derived from lesser omentum: case report. Medicine (Baltimore).

[3] Melo IA, Camargo JJ, Gomes BM, Cabrera GA, Machuca TN (2009). Isolated mediastinal cystic lymphangioma. Rev Port Pneumol.

[4] Bhardwaj R, Vaziri H, Gautam A, Ballesteros E, Karimeddini D, Wu GY (2018). Chylous ascites: a review of pathogenesis, diagnosis and treatment. J Clin Transl Hepatol.

[5] Fernandes FF, Alves VO, Sánchez TE, Paula WD, Santana AN (2016). Chylothorax in paracoccidioidomycosis. Rev Inst Med Trop São Paulo.

[6] Javier Uribe M, Rolando Sepúlveda C, Rodrigo Cruz N (2018). Chylous ascites management: clinical case and literature review. Gastroenterol Latinoam..

[7] Ghimire S, Shah H, Paudel S, Yang TJM, Khan HM (2020). Chylous ascites and pleural effusion treated with intravenous Octreotide. Cureus.

[8] Oré Acevedo JF, La Torre Caballero LM, Urteaga Quiroga RL (2020). Surgical treatment of lymphatic malformations in pediatric patients. Acta Otorrinolaringol Cir Cabeza Cuello..

[9] Gomes CS, Handa GI, Silveira FP, Buzingnani VZ, Binati FM, Rasera ESL (2009). Tratamento cirúrgico da ascite quilosa. J Vasc Bras.

[10] Pessotti CFX, Jatene IB, Buononato PEU, Elias PF, Pinto ACD, Kok MFJ (2011). Uso do octreotide no tratamento do quilotórax e quiloperitôneo. Arq Bras Cardiol.

[11] Parthasarathy S, Shah S, Raja Sager A, Rangan A, Durugu S (2020). Staphylococcus lugdunensis: review of epidemiology, complications, and treatment. Cureus.

